# Instructional changes instigated by university faculty during the COVID-19 pandemic: the effect of individual, course and institutional factors

**DOI:** 10.1186/s41239-021-00286-7

**Published:** 2021-09-21

**Authors:** Jihyun Lee, Insung Jung

**Affiliations:** 1 Department of Dental Education, School of Dentistry and Dental Research Institute, Seoul National University, 103 Daehak-ro, Jongno-gu, Seoul, 03080 Republic of Korea; 2 Department of Education and Language Education, International Christian University, Tokyo, Japan

**Keywords:** COVID-19 pandemic, Ecological systems, Emergency online teaching, Faculty innovativeness, Instructional change, Technology adoption, University educators

## Abstract

The purpose of this study was to investigate instructional changes made by faculty for emergency online teaching necessitated by the COVID-19 pandemic, and hence to explore key factors related to those changes from an ecological systems perspective. Data on various individual, course, and institutional factors and instructional change variables were collected from 201 educators at higher education institutions. Results revealed that the level of instructional changes made by faculty was on average between substituting their existing course for an online one with some functional improvement (augmentation-level 3) and critical course redesign (modification-level 4), but that educators did not reach the level of the creation of new tasks which were previously inconceivable (redefinition-level 5). The biggest instructional change was found to be in teaching behaviors, followed by technology use, with only small changes in beliefs about online teaching. Factors that most highly correlated with instructional change were individual educators’ technology acceptance and innovation propensity, media synchronicity of the course, and the fidelity of institutional support. Recommendations are provided to aid strategic coping by universities facing a major crisis, with insights that may ultimately improve the quality of higher education in non-crisis contexts.

## Introduction

With the rapid growth of online education, many researchers have explored experiences of faculty online teaching, including occurring during crises, such as natural disasters, or socio-political turmoil. In interviews with eight teachers who introduced online technology during the spread of the SARS virus in Hong Kong in 2003, Fox (2007) found that teachers saw both the potential and shortcomings of using online technology in education, with the negative impacts due mostly to lack of preparation time and teaching competency. After studying online teaching in the wake of Hurricane Katrina, Lorenzo (2008) concluded that higher education is often slow to adopt new tools and innovations and institute changes accordingly; when there is a will to do so even during a crisis. Ayebi-Arthur (2017), in a study of nine faculty members in New Zealand who offered online teaching after the earthquakes of 2010–2011, revealed that while most faculty members perceived the usefulness of online learning during the crisis, frequent communication, technology infrastructure and support were the keys to faculty successfully adopting online tools. Further, in a case study of academics who taught blended and online learning courses during student protests in South Africa between 2015 and 2016, Czerniewicz et al. (2019) found that most faculty accepted blended and online approaches as viable options during the university shutdowns, although face-to-face teaching as the more effective and preferred method of instruction.

In a similar vein, a few studies have investigated emergency online teaching by faculty during the COVID-19 pandemic that began in late 2019 and continues today. In a survey of U.S. faculty and administrators from 672 institutions, conducted in the early weeks of emergency online teaching, Johnson et al. (2020) discovered that regardless of whether faculty had taught online before, they were able to quickly adopt online teaching approaches and make the necessary adjustments to assignments, exams, and grading policies. Similar findings to these can be observed in studies conducted in less developed countries as well. In a qualitative study based on interviews with 20 professors from Indian universities, Shenoy et al. (2020) found that even those who initially resisted the adoption of technology and perceived online technology as a hindrance, quickly developed habits conducive to teaching online, utilizing appropriate tools and in time perceiving technology adoption to be a blessing and a welcome revolution in instruction. Similarly, Almaghaslah and Alsyari (2020) found that of a surveyed 59 Saudi Arabian faculty members the majority reported a smooth shift from classroom teaching to emergency online instruction and expressed an appreciation for the flexibility of the latter approach. A recent auto ethnography study conducted in Japan (Jung et al., 2021) also revealed that faculty members generally became more optimistic and utilized more diversified resources in emergency online teaching with more online teaching experience. All of these studies of faculty experiences during the COVID-19 pandemic are informative, as they reveal faculty members’ ability to quickly adopt online technologies and adaptation to emergency online teaching, as well as their confusion, anxiety, and struggles in the early stages of the transition. What these studies fail to explore, however, are the factors associated with the rapid adoption of new technologies and adjustments to emergency online teaching by faculty.

Several theories and models, such as Social Cognitive Theory, the Technology Acceptance Model, the Theory of Planned Behavior, and the first and second Unified Theory of Acceptance and Use of Technology (UTAUT), have identified key factors that may explain why individuals adopt certain technologies or innovations (Jung & Lee, 2020). For instance, the Technology Acceptance Model predicts technology acceptance based on the three factors of perceived usefulness, perceived ease of use, and user acceptance of information technology (Davis, 1989). Many researchers have used these factors to examine the acceptance of various technologies, including those involved in online teaching in higher education contexts (e.g., Alsofyani et al., 2012; Gibson et al., 2008; Green et al., 2009; King & He, 2006; Yuen & Ma, 2008). Although the robustness of the Technological Acceptance Model is well supported, criticisms of it include its exclusion of social influence and individual features (Ajibade, 2018). The UTAUT presents a viable alternative in that it uses four factors: performance expectancy, effort expectancy, social influence, and facilitating conditions, to both explain technology acceptance and acknowledge the moderating effects of age, gender, and experience (Venkatesh et al., 2003). Like the Technology Acceptance Model, the Unified Theory of Acceptance and Use of Technology has been widely applied in examining faculty members’ acceptance of online teaching across different contexts (Gunasinghe et al., 2019a; Hu et al., 2020; Radovan & Kristl, 2017).

Over the past few decades, there have been several studies which have examined factors influencing faculty adoption of online education. Some of these studies reveal faculty members are motivated to teach online when incentives at institutional level are offered, including but not exclusively, existence and quality of institutional support (Alsofyani et al., 2012; Moore & Anderson, 2003; Sumrall, 2002), which may include institutional commitment to online education and university policy to create supporting and facilitating environments for online education (Gannon-Cook & Ley, 2004; Theall, 1999), and personal rewards such as time and monetary compensation (Gannon-Cook & Ley, 2004; Townsend & Hauss, 2002). Other studies report that individual characteristics of faculty such as age, gender, teaching experience and intrinsic motivation (e.g., personal growth, satisfaction and experience gained from teaching online) influence acceptance and continuation of teaching online (Allan & Seaman, 2012; Chapman, 2011; Ko & Rossen, 2003; Shea, 2007). Granic and Marangunic (2019), Thatcher et al. (2007) and Agarwal and Prasad (1998) argue that personal innovativeness or innovation propensity influence individuals’ technology acceptance. Yet, other studies find that course-level instructional design and media factors, such as flexibility, collaborative discussions, and multimedia components affect faculty adoption of online education (Arend, 2009; Chapman, 2011). During a crisis like an earthquake or indeed COVID-19, some studies like that of Almaiah et al. (2020) and Ayebi-Arthur (2017) point out that an institution’s policies and strategies to help its faculty members readily cope with the crisis in question are influential in facilitating the implementation of online education.

While the technology and innovation adoption theories and relevant empirical studies may help researchers better understand factors influencing adoption behaviors across diverse contexts, their focus is mainly on individuals' voluntary use of a technology or their acceptance of an innovation including online teaching, when such opportunities exist (Dillon, 2001; Gunasinghe et al., 2019b; Taherdoost, 2018). These theories are therefore only able to offer limited predictive capabilities for those situations in which individuals have no choice other than to pursue the same end within more or less the same time and resource framework. Such is the case in emergency online teaching situations, where individual faculty members are obliged to develop and implement emergency online courses, using certain available technologies and in accordance with certain requirements specified by their university. Even when all faculty members are mandated to adopt online teaching, they of course make technological and pedagogical choices concerning what media or technology they will use and what instructional practices they will implement. To examine the factors affecting such instructional changes during emergency online teaching, one must consider the social processes that take place between the individual and the surrounding contexts as well as other individual and organizational factors. In this regard, the present study applies, as an umbrella framework, Bronfenbrenner’s ecological systems theory, with its focus on the relationship between individual experience and interactions with surrounding contexts, and further, draws on the Technology Acceptance Model to identify factors affecting instructional changes made by university faculty members providing emergency online teaching during the COVID-19 pandemic.

### Theoretical framework

The ecological systems theory, developed by Bronfenbrenner (1977, 1979), served as the theoretical framework for this study. The theory explains how the intrinsic qualities of individuals and their immediate and broader environments interact to influence how they develop and change. Bronfenbrenner’s theory organizes environments into four systems with which an individual interacts (Bronfenbrenner, 1977, pp. 514–515): the micro-system, meso-system, exo-system, and macro-system.

- The most proximal system to the individual is the *micro-system,* which refers to the immediate setting where one exists and usually performs a role or activity.
- Moving outward from the microsystem is the *meso-system*, which incorporates all of one's micro-systems and the processes and interactions between them.
- The *exo-system* is the next outermost system, which contains elements of one’s environment that one cannot control or effect, often including one’s neighborhood and other social and societal structures.
- The outermost system is the *macro-system,* which includes one’s culture or subculture and, in some cases, one’s socio-economic setting, race, and country.

In the present study, the *individuals* in Bronfenbrenner’s formulation were South Korean university faculty members who have been obliged to carry out emergency online teaching during the COVID-19 pandemic. Individual factors that were found to be influential in the previous studies were also found to be present in this study: technology acceptance and innovation propensity (Agarwal & Prasad, 1998; Granic, & Marangunic, 2019; Stewart et al., 2010; Thatcher et al, 2007), and teaching perspective (Wingo et al., 2017) along with their gender, age and teaching experience (Allan & Seaman, 2012; Shea, 2007). The immediate setting or *microsystem,* with which each faculty member interacted, was a course they were required to teach online as a result of the pandemic. It included such factors as instructional objectives, media, and instructional design (Arend, 2009; Chapman, 2011) along with course disciplinary area, course level and class size. The *meso-system* was the institution in which faculty taught, and included such factors as the size, type, and location of the university (Green et al., 2009), the fidelity of institutional support (Alsofyani et al., 2012; Sumrall, 2002), and organization-level upheaval and nudge strategies employed to cope with the crisis (Almaiah et al., 2020; Ayebi-Arthur, 2017). Factors that were found to be important, mostly for voluntary decisions to accept a technology or online education, such as personal rewards were not included in the study as the context of this present study was in online education as mandatory requirements. The *exo-system* (e.g., government and national strategies and policies) and *macro-system* (e.g., national culture) were also not included in the research design, since they were the same across the board for all faculty members involved.

**Fig. 1:**
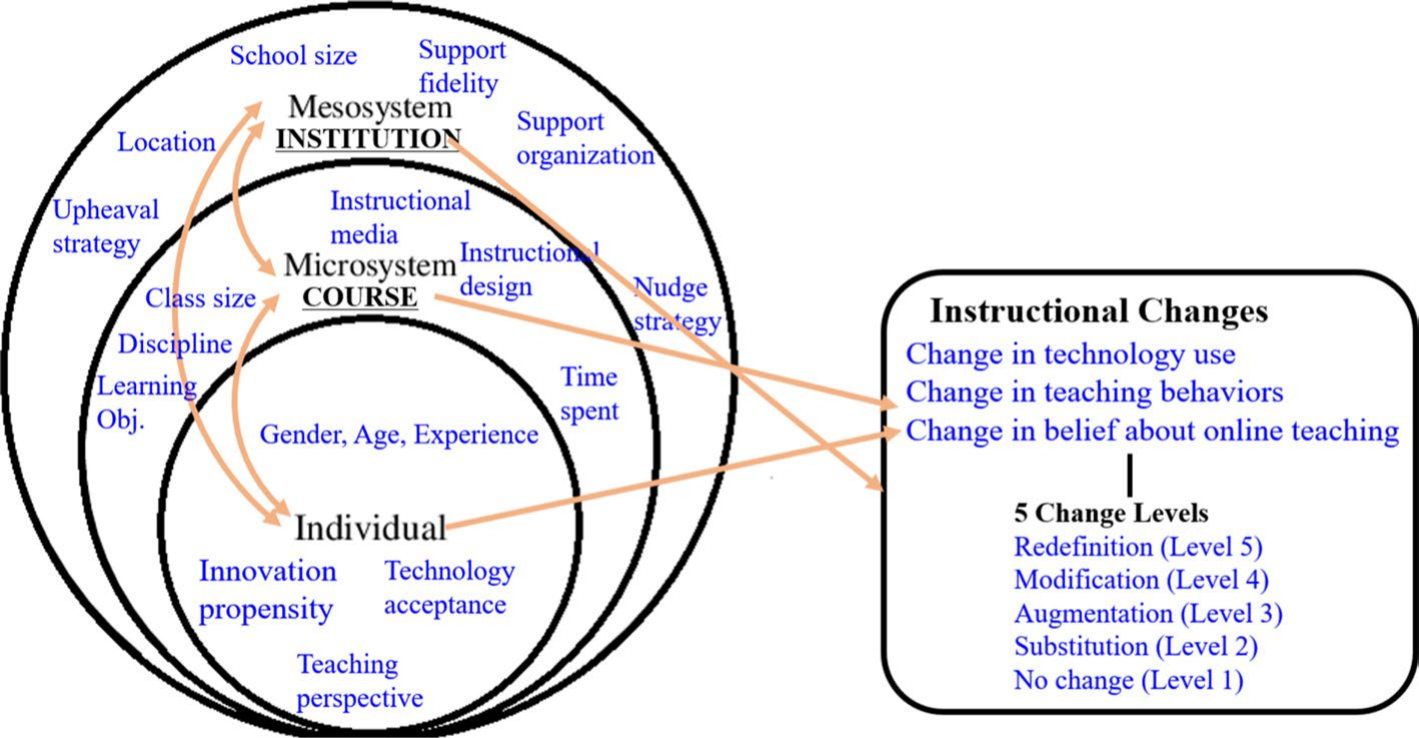
Theoretical framework of the study

Keeping in mind the theoretical notions about individuals and micro and meso-systems of the ecological systems model, we hypothesized that factors at three different system levels: the individual, course, and institutional level interact with each other to affect the instructional changes instigated by faculty, in emergency online teaching. Specifically considered were changes in technology use, teaching behaviors, and beliefs about online teaching in five levels (Fig. 1).

### Research questions

We applied our theoretical framework in developing the following research questions:

1. To what extent did university faculty change their emergency online teaching during the COVID-19 pandemic in terms of: (1) technology use, (2) teaching behaviors, and (3) beliefs about online teaching?
2. How did factors at individual, course, and institutional levels contribute to the changes that faculty instituted in their emergency online teaching?

## Methods

### Participants and data collection

A total of 201 university educators at higher education institutions, located in South Korea, participated in this study. The sample comprised 58.26% (n = 117) women, 40.8% (n = 82) men, and 1% (n = 2) who preferred not to state their gender. In regard to age, 6.0% (n = 12) of participants were in their thirties, 39.8% (n = 80) in their forties, 43.3% (n = 87) in their fifties, and 10.9% (n = 22) in their sixties. Teaching experience was relatively evenly distributed, with 8.5% (n = 17) with less than 5 years of teaching experience, 23.9% (n = 48) 6 to 10 years, 20.4% (n = 41) 11 to 15 years, 15.9% (n = 32) 16 to 19 years, and 31.3% (n = 63) more than 20 years. Most participants (80.6%, n = 162) could be considered to be at the beginner level of online teaching, with most at this level (69.2%, n = 139) having with *no experience prior to COVID-19* and a smaller number at this level (11.4%, n = 23) having *less than one-year* of online teaching experience. Among the participants, 56.2% (n = 113) taught courses in the arts, humanities, and social sciences, 36.8% (n = 74) taught courses in engineering, sciences, and medical disciplines, and 7.0% (n = 14) taught multi-disciplinary courses. Course sizes were diverse: 3.0% (n = 6) of courses contained less than 10 students, 40.8% (n = 82) of 10 to 30 students, 31.8% (n = 64) of 31 to 50 students, 19.4% (n = 39) of 51 to 100 students, and 5.0% (n = 10) of more than 100 students. Institution sizes ranged from less than 10,000 students (62.7%, n = 126), to more than 10,000 students (37.3%, n = 75).

Data collection was from May 21, 2020 to July 2, 2020 using an online survey. The study design was approved by the Research Ethics Committee of the higher education institution of one of the authors (#2020-09). All study participants provided informed consent on the first page of the survey.

### Measurement

The theoretical framework shown in Fig. 1, above, was used for the development of an initial survey and the conducting of two pilot tests with five university educators in an effort to detect erroneous or unclear expressions or any parts that might lead to biased responses. The final version consisted of 63 items across four sections designed to measure: (1) individual factors (23 items), (2) course factors (17 items), (3) institutional factors (20 items), and (4) instructional change variables (3 items).

*Individual factors* The section of the survey measuring individual factors consists of four sub-parts: (1) background information on gender, age, teaching experience and online teaching experience, (2) innovation propensity, (3) technology acceptance, and (4) teaching perspectives. To measure innovation propensity, we adapted 10 items from a survey by Savery (2005) that was developed based on Rogers’ diffusion of innovations theory, which categorizes individuals as innovators, early adopters, early majority, late majority, or laggards based on their behavior in relation to innovations, especially those involving technology. The total innovation propensity score was calculated by combining the scores of innovators and early adopters with the scores of those in the reverse-coded early majority, late majority, and laggards categories. The internal consistency of innovation propensity was 0.729. In regard to technology acceptance, four constructs were measured (perceived ease of use, perceived usefulness, attitude, and enjoyment) using nine items from a survey by Park (2009) modified to fit the context of this study. The internal consistency of technology acceptance was 0.853. To measure teaching perspectives, participants were asked to rate the importance of each of the roles of lecturer, designer, and facilitator for successful online teaching. The internal consistency of the three items that measured teaching perspectives was 0.756. The overall consistency of the individual factors, excluding individual background items, was 0.854.

*Course factors* The section of the survey measuring course factors consists of four sub-parts: (1) background information on course discipline, course size, and course objectives, (2) media synchronicity, (3) the extent of modification of the previous course, and (4) the time the educator spent implementing the course. In order to measure the extent to which faculty used synchronous media in the course three questions were posed about the use of synchronous media for content delivery, faculty-student interaction, and student–student interaction. The internal consistency of the media synchronicity items was 0.808. To measure the extent of modifications required to convert the course to an online format, five items were employed asking about modifications in learning content, instructional strategy, assessment, and interactions. The internal consistency of these items was 0.724. As for time spent on the course, the participants were asked to estimate the time and energy they spent conducting technology-related tasks, preparing lectures, implementing lectures, giving feedback or answering students’ questions, facilitating or motivating student learning, and conducting assessments. The internal consistency of the six items on time spent on the course was 0.813. The overall consistency of course factors, excluding background items, was 0.762.

*Institutional factors* The section of the survey on institutional factors consists of four sub-parts: (1) background information on the size and location of the institution and the support it offers, (2) the fidelity of support the institution provided, (3) nudging strategies (strategies to influence behavior without restricting options or incentives) (Thaler & Sunstein, 2008), and (4) upheaval strategies (strategies that nations, institutions, or individuals adopt to manage existential crisis). To measure the fidelity of institutional supports for teaching online, the participants were asked to rate the degree of helpfulness of five different forms of support: (1) face-to-face training, (2) online training, (3) text guidelines, (4) video tutorials, and (5) hot line or consultation. The internal consistency of these five items was 0.812. To examine nudging strategies adopted by the institution during the pandemic, four questions adapted from Thaler and Sunstein (2008) were asked. The internal consistency of these items was 0.645. Eight items taken from Diamond’s (2019) twelve successful upheaval strategies in the context of COVID-19 pandemic were asked to identify upheaval strategies of the institution. The internal consistency of these items was 0.924. The overall consistency of the items for the course factors, excluding course background items, was 0.903.

*Instructional changes* The section of the survey on instructional changes consists of three items measuring: (1) changes in technology use for courses being transitioned from a traditional course to an emergency online course, (2) changes in teaching behavior, and (3) changes in beliefs about online teaching. The possible responses for degree of change included the four levels of Puentedura’s (2006) Substitution-Augmentation-Modification-Redefinition (SAMR) model, with an additional one added for ‘no change’. The consistency of the three items measuring instructional change was 0.869.

- No change (Level 1): online teaching with no change from classroom face-to-face teaching
- Substitution (Level 2): online teaching as a direct tool substitute with little functional change
- Augmentation (Level 3): online teaching as a direct tool substitute with functional improvement
- Modification (Level 4): online teaching as a tool for significant task redesign
- Redefinition (Level 5): online teaching as a tool for the creation of new tasks, previously inconceivable

### Data analyses

To address the posed research questions, descriptive statistics, bivariate correlation analysis, independent and paired samples t-tests, and analyses of variance (ANOVA) were employed. To test the hypotheses in the research model shown in Fig. 1, variance-based structural equation modeling was conducted, employing the partial least squares approach, PLS-SEM using SmartPLS 3 software (SmartPLS GmbH, Germany, 2017). PLS-SEM was chosen as it is considered more relevant for analyzing complex models and exploratory research (Hair et al., 2017). Calculated descriptive statistics and correlations provided the preliminary data examination. The second-order measurement model were assessed, followed by the structural model: assessment of the measurement model required checking internal consistency reliability using the Cronbach alpha and composite reliability, convergent validity using outer loadings and Average Variance Extracted (AVE), and discriminant validity using cross loadings and Fornell-Larcker criteria. Based on the results of the measurement model assessment, indicators that were below the recommended thresholds were deleted and the model refined, and measurement validation for the new model conducted. All constructs in the resulting measurement model had acceptable reliability and validity (all Cronbach’s alphas greater than 0.6, composite reliability between 0.6 and 0.9, and AVE for constructs higher than 0.5). Structural relationships among individual factors, course factors, institutional factors, and instructional change variables were validated through a bootstrap analysis with 500 adjusted samples that approximated distribution. In assessing this structural model, we checked the collinearity statistics and calculated the path coefficients along with *R*^*2*^, *F*^*2*^, and *Q*^*2*^ to measure the quality of the model.

## Results

### Instructional changes made by faculty during the COVID-19 pandemic

Firstly, the mean and standard deviations of three instructional change variables; faculty changes in the use of technology, teaching behaviors, and beliefs on online teaching, as well as the total average change of all three (Table 1) were calculated. Faculty change in teaching behaviors had the highest mean (*M* = 3.53, *SD* = 1.02) and, at a similar magnitude, faculty change in the use of instructional technology had the next highest mean (*M* = 3.51, *SD* = 1.11). The mean of the change in faculty beliefs about online teaching was significantly lower (*M* = 3.45) than the mean of technology change [*t*(200) = 3.61^***^] and behavior change (*t*(200) = 2.73^**^), and had the largest variance among the other instructional change variables (*SD* = 1.18). The three change variables were significantly and positively related to each other (*β* = 0.63 ~ 76), and the total average change for all three variables was 3.45, equivalent to 69.1% of the perfect level.

**Table 1:** Descriptive statistics of instructional change variables and mean differences by background variables of individual, course, and institutional factors

Change in	M	SD	Correlation			Mean Differences									
						Individual factors				Course factors			Institutional factors		
			1	2	3	Gender	*Age*	*Teaching Exp*	*Online teach Exp*	*Course discipline*	*Course size*	*Course Obj*	*Inst. Size*	*Loaction*	*Support org*
1. Media/material	3.51 (70.1%)	1.02	(1)	0.68^**^	0.63^**^	F > M *t*(197) = − 2.2^*^	*n.s*	*n.s*	*n.s*	*n.s*	*n.s*	*n.s*	*n.s*	*n.s*	*n.s*
2. Practice/behavior	3.53 (70.5%)	1.11		(1)	0.76^**^	*n.s*	*n.s*	*n.s*	*n.s*	*n.s*	*n.s*	*n.s*	*n.s*	*n.s*	*n.s*
3. Attitude/Understanding of teaching and learning	3.32 (66.5%)	1.18			(1)	*n.s*	*n.s*	*n.s*	*n.s*	*n.s*	N9 < N10, N30, N50, N100 *F*(4,196) = 3.75^**^	*n.s*	*n.s*	*n.s*	*n.s*
Change total	3.45 (69.1%)	0.98				*n.s*	*n.s*	*n.s*	*n.s*	*n.s*	*n.s*	*n.s*	*n.s*	*n.s*	*n.s*

*n.s* no significant difference, ***p < 0.001, **p < 0.01, *p < 0.05, and *n.s.* *p* > 0.05

*F* female faculty members, *M* male faculty members, *N9* class size with less than 10 students, *N10* class size with 10–30 students, *N30* class size with 31–50 students, *N50* class size with 51–100 students, and *N100* class size more than 100 students

Secondly, independent sample t-tests and one-way between-subjects analyses of variance (ANOVA) were performed with a view to investigating whether the total instructional change differed by background factors at the *individual* level (gender, age, teaching experience, and online experience of faculty members), *course* level (the discipline area of the course, the number of students taking the course, and the learning objectives the course aims for) and *institutional* level (the number of students in the university the faculty member is working for, the geographic location of the institution, and whether the institution has an organization that supports faculty teaching during COVID-19). No statistically significant difference in the total change for any of the background factors was found.

The two background factors of gender and class size were associated with differences in some instructional change variables, however. Change in technology use was significantly higher for female faculty members (*M* = 3.65, *SD* = 1.02) than for their male counterparts (*M* = 3.33, *SD* = 0.98) with a medium value of effect size [*t*(197) = − 2.2, *p* = 0.03, *d* = 0.32]. Change in beliefs about online teaching was significantly lower for courses with fewer than 10 students (*M* = 1.83, *SD* = 0.98) compared to courses with 10 to 30 students (*M* = 3.48, *SD* = 1.19), 31 to 50 students (*M* = 3.14, *SD* = 1.13), 51–100 students (*M* = 3.41, *SD* = 1.16), and more than 100 students (*M* = 3.80, *SD* = 1.18), with a large effect size (*F*(4, 196) = 3.75, *p* = 0.006, *η*^*2*^ = 0.07). The differences between courses with more than 10 and fewer than 100 students were not significant (*p* = 0.20) also, none of the institutional background factors made a significant difference in instructional change.

Thirdly, bivariate correlation analyses was conducted among particular individual factors, specifically; innovation propensity, technology acceptance, and teaching perspectives of faculty members, course factors; the degree of synchronicity of the media used in the course, amount of modification from the previous course, and the time and effort faculty members spent modifying the course, and institutional factors; the fidelity of instructional support provided by the institution, the extent of nudging strategies, and the amount of upheaval strategies used by the institution.: also calculated were the correlations between those factors and instructional changes, Table 2, below. The results show that most of the sub-factors, with each factor, were significantly related to each other. Among the individual factors, technology acceptance had the strongest correlation to instructional change variables (*r* = 0.37, 0.37, 0.36, and 0.41 for change in media/technology, practice/behavior, attitude/understanding of teaching and learning, and total change respectively, all *p* = 0.00). Among course factors, media synchronicity was highly correlated to total change (*r* = 0.35, *p* = 0.00), and among institutional factors, the fidelity of instructional support was correlated to change in media/technology use of educators (*r* = 0.29, *p* = 0.00).

**Table 2:** Descriptive statistics of individual, course, and institutional factors and correlation to instructional change variables

	1.1	1.2	1.3	2.1	2.2	2.3	3.1	3.2	3.3	Correlation to instructional changes			
										Change in media/material	Change in practice/behavior	Change in attitude/understanding of teaching and learning	Change total
1.Individual factors													
1.1 Innovation propensity	(1)	0.23^***^	0.05	0.16^*^	.13	0.06	0.07	0.19^**^	0.53	0.21^**^	0.17^*^	0.15^*^	0.20^**^
1.2 Technology acceptance		(1)	0.08	0.33^**^	0.38^**^	0.17^*^	0.24^**^	0.18^**^	0.13	0.37^**^	0.37^**^	0.36^**^	0.41^**^
1.3 Teaching perspectives			(1)	0.19^**^	− 0.01	0.18^*^	− 0.02	0.03	0.03	0.00	0.17^*^	0.15^*^	0.12
2. Course factors													
2.1 Media synchronicity				(1)	0.27^**^	0.27^**^	0.22^**^	0.05	0.10	0.25^**^	0.34^**^	0.34^**^	0.35^**^
2.2 Amount of modification					(1)	0.05	0.10	0.12	0.13	0.14	0.07	0.13	0.12
2.3 Time spent for the course						(1)	0.16^*^	0.03	0.07	0.26^**^	0.26^**^	0.21^**^	0.27^**^
3. Institutional factors													
3.1 Institutional supports fidelity							(1)	0.30^**^	0.54^**^	0.29^**^	0.19^**^	0.20^**^	0.25^**^
3.2 Nudging strategies								(1)	0.53^**^	0.17^*^	0.15^*^	0.06	0.14^*^
3.3 Upheaval strategies									(1)	0.26^**^	0.16^*^	0.11	0.20^**^
M	3.98	3.16	3.88	3.30	2.56	3.99	2.90	3.55	3.77	3.51	3.53	3.32	3.45
SD	0.50	0.74	0.74	1.13	0.76	0.65	1.15	0.68	0.82	1.02	1.11	1.18	0.98

***p < 0.001, **p < 0.01, and *p < 0.05

### Factors affecting instructional changes by faculty during the COVID-19 pandemic

**Fig. 2:**
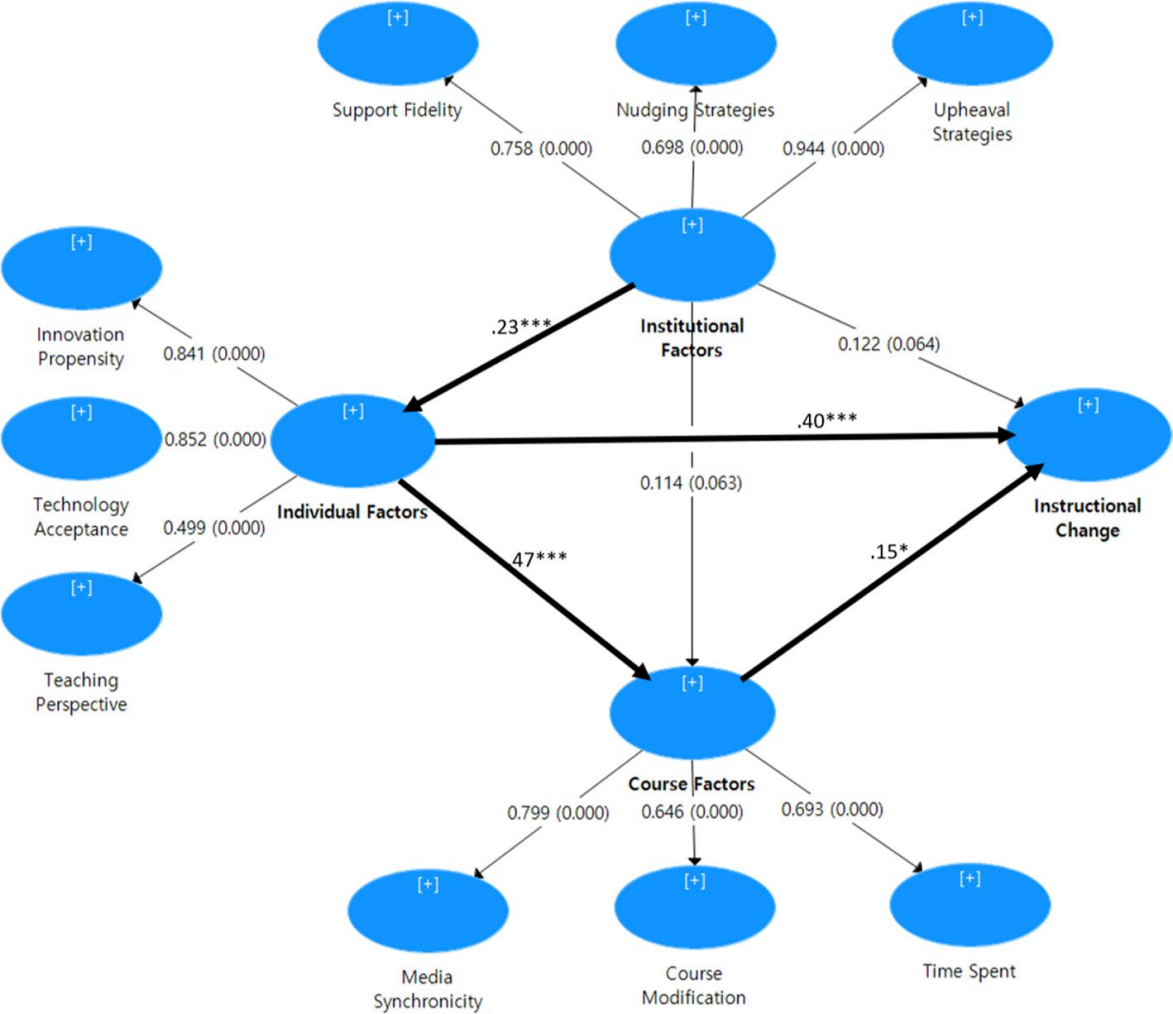
Path coefficients of the research model

To assess both the measurement model and the structural model PLS-SEM was applied. In assessing the structural model, path coefficients and their significance were examined (Table 3 and Fig. 2) and found to be all significant except for the paths from institutional factors to course factors (*p* = 0.063) and from institutional factors to instructional change (*p* = 0.064), suggesting that institutional factors do not lead to instructional change directly, but via individual factors, and not via course factors. The strongest paths were found from individual factors to course factors (*β* = 0.47, *p* = 0.000) and to instructional change (*β* = 0.40, *p* = 0.000), followed by the path from institutional factors to individual factors (*β* = 0.23, *p* = 0.000). The path from course factors to instructional change was also significant, but with a small effect size (*β* = 0.15, *p* = 0.041). The model explains 29.3% of the variance in instructional change by faculty members during the COVID-19 pandemic, which, in education research, is indicative of significant explanatory power (*R*^*2*^ = 0.293, *Adj R*^*2*^ = 0.282) (Eisenhauer, 2009), and has acceptable predicative relevance value with a *Q*^*2*^ exceeding zero (*Q*^*2*^ = 0.26) (Hair et al., 2017).

**Table 3:** Results of structural model assessment

Independent variables	Dependent variables	Standardized causal effects			*T*-statistics	*f*^2^ Effect size
		Direct	Indirect	Total		
Individual factors	Course factors	0.47		0.47	8.80^***^	0.29
	Instructional change	0.40	0.07	0.47	6.25^***^	0.17
Course factors	Instructional change	0.15		0.15	1.97^*^	0.02
Institutional factors	Individual factors	0.23		0.23	3.55^**^	0.06
	Course factors	0.11	0.11	0.22	1.90 ^n.s^	0.02
	Instructional change	0.12	0.13	0.25	1.87 ^n.s^	0.17

***p < 0.001, **p < 0.01, and *p < 0.05

## Discussion

The purpose of this study was to investigate the instructional changes made by faculty for emergency online teaching necessitated by the COVID-19 pandemic, as well as the key factors influencing those changes.

The results indicate that educators made the most drastic changes in their teaching behaviors, followed by changes made to their use of technology. The change in their beliefs about online teaching was small but significant. This finding is contrary to what one might expect given classical theories such as the Theory of Planned Behavior (Ajzen, 1985), the Technology Acceptance Models (Davis, 1989; Venkatesh, 2000, Venkatesh et al., 2003, 2012), the Information-Motivation-Behavioral Skills Model (Fisher & Fisher, 1992), and the Transtheoretical Model of Change (Prochaska & DiClemente, 1983), which claim that changes in beliefs and attitudes inevitably precede changes in behavior. Our contradictory finding may be due to the unique context of this study, where changes in teaching behaviors and technology adoption were urgently required for emergency online teaching even if attitudinal and belief changes had not occurred. Several studies (Ertmer et al., 2001; Fang, 1996; Farrell & Lim, 2005) have indicated that context, including the school/classroom/course context and the policy context, is an important factor affecting inconsistencies between teacher beliefs and practices. The unavoidable context of the COVID-19 pandemic has likely forced many educators to change their teaching behavior without making corresponding changes in their beliefs. As asserted by Festinger (1957), such a belief-behavior inconsistency would create cognitive dissonance for educators, whose natural human drive for cognitive consistency would motivate them to change their beliefs to justify the behavior they are required to perform (e.g., Cooper, 2007; Festinger & Carlsmith, 1959). Research on educators’ behavioral and attitudinal changes in various contexts is needed to test this as yet unproven argument.

Overall, the change identified in this study represents about 70% of perfect level (M = 3.45 out of 5.00). According to the SAMR model, this level corresponds to the relationship between Augmentation (level 3.0) and Modification (level 4.0); that is, university educators, on average, converted their existing courses to online courses with some functional improvements (Augmentation) and a modest revision of critical course redesign components (Modification), without quite reaching the level of creating new tasks that were previously inconceivable (Redefinition 5.0). This level of change may be regarded as a substantial outcome given that educators were required to create online courses in a short period of time and without much preparation or assistance. What, then, hindered educators from advancing to the Redefinition stage? According to Christensen (2003), innovation in business is of two types: (1) sustained innovation, which includes changes but does not affect an existing market, and (2) disruptive innovation, which creates a new market with a new set of values, ultimately overtaking an existing market. Disruptive innovation may correspond to Redefinition stage of the SAMR model, which involves radical and structural changes in existing practices. To reach the Redefinition stage and bring about disruptive innovation in online teaching, educators need to develop a high level of competency. They must understand the affordances of new technologies and be able to apply this knowledge to create new online pedagogies. It is to be hoped that further research may delve into potential factors that can bring about redefinition or disruptive changes.

This study also revealed that at individual, course, and institutional levels, background factors such as age, teaching experience, online experience, discipline, institution size, geographic location, and supporting organization, do not affect instructional change. Gender, however, appears to help explain, in part, the change in technology use. Our findings, that female faculty made greater changes in technology use in the context of emergency online teaching, may suggest their use of technology in their pre-COVID-19 teaching was less than their male counterparts, requiring more active change in technology adoption in emergency online teaching. Gender differences in technology use have been reported in other studies (e.g., Kahveci, 2010; Yau & Cheng, 2012), where researchers found that males had a higher level of confidence in and usage of technology compared to females. Some recent research (e.g., Gebhardt et al., 2019; Guillén-Gámez et al., 2020) suggests that this gender gap is becoming less evident, however, with the increased prevalence and importance of digital technologies in society and in higher education. Further investigation into gender differences in faculty technology use would help understand inconsistencies between these findings and previous research. Another possible interpretation of gender differences in this study may be that female educators respond to forced change differently and are more compliant with institutional-level decisions than their male counterparts, which suggests future research through in-depth qualitative research.

Class size, another course background sub-factor, was significant in explaining changes in beliefs concerning online teaching. Contrary to the assertions of numerous studies; that a small class size is more beneficial for quality teaching (e.g., Blatchford et al., 2003), this study shows that faculty are more likely to change their beliefs about online teaching when teaching a class of between 10 and 100 students: while those with less than 10 students in their class reported less change in their beliefs about online teaching. Explanation for this could be the observation that course preparation, instructional design, or instructional changes may be more influential in relatively large-sized courses compared to small-sized ones, eventually leading to greater changes in faculty beliefs and understandings about online teaching in larger classes (Elison-Bowers et al., 2011).

While most individual, course, and institutional background factors were not influential in explaining the instructional changes of faculty faced with emergency online teaching, some did show a degree of impact. Among them, technology acceptance and innovation propensity (individual factors) had two of the strongest associations with instructional change, followed by the fidelity of institutional support (institutional factor) and media synchronicity (course factor). Technology acceptance and innovativeness in individual educators have long been recognized as critical prerequisites for technology integration into teaching (e.g., Granic, & Marangunic, 2019). In a study of 237 primary and secondary school teachers, Akar (2019) concluded that teachers’ personal innovativeness affects their technology acceptance in teaching. Similarly, in a study of 92 university educators, Akgün (2017) found that educators’ individual innovativeness features and technology acceptance influenced their intention to use technology in instruction. Consistent with previous findings, then, this study confirms the importance of helping faculty develop technology acceptance and innovativeness in order to pave the way for resiliency when changes in modes of teaching are required.

At the institutional level, the fidelity of instructional support provided by the institution was another factor strongly associated with instructional change, particularly in regard to change in technology use. Instructional support of this sort may have some equivalencies to the ease of use in Technology Acceptance Model, behavioral control in Theory of Planned Behavior (TPB), and facilitating conditions in UTAUT. In other words, proper instructional support, provided by an institution, can assist educators in making use of technology more easily, flexibly, and with greater facilitation, regardless of whether the behavior is voluntary or obligatory. Ayebi-Arthur (2017) reported that one of the most important factors that helped overcome the 2011 earthquakes in a New Zealand university was institutional support for faculty in the use of e-learning technologies, along with the availability of technological tools where the former induced ease of use of the tools.

Interestingly, the synchronicity of instructional media used in the course being correlated with instructional change might be explained by media synchronicity theory, which holds that synchronous media are more effective for verifying, adjusting, or negotiating participants’ mental models so as to enhance shared understanding. Synchronous media can be more beneficial to lead instructional change than asynchronous media because instructors are able to receive learners’ reaction and feedback more directly and readily, in real time, while interacting with them to improve their mental models (Dennis et al., 2008). Moreover, this result has a cultural perspective interpretation: in a high-context communication culture like Korea, where in-person interaction is highly valued, synchronous, visual technologies tend to be more readily accepted than asynchronous, text-based tools, since the former simulate the face-to-face presence (Jung & Gunawardena, 2014).

Finally, the structural equation modeling that examined the path from individual, course, and institutional factors to instructional change, provided several insights. It revealed that individual factors reflecting progressive tendencies (e.g., technology acceptance and innovation propensity) had the most powerful influence over course factors (e.g., media selection and time and effort for the course) eventually leading to instructional change. Conversely, institutional factors had no direct impact on the instructional change or course factors. These findings point to the importance of faculty innovativeness and technology acceptance in bringing about changes in teaching and learning, especially during a crisis, as Everett Rogers posited. Rogers’ diffusion of innovations theory put individual adopters at the center of innovation diffusion, before the later addition of organizational and social influences (Rogers, 2003).

In this study, within its unique context of urgent societal change, such progressive tendencies among individual faculty members, when facilitated by institutional supports and strategies, were a critical driver in the instructional change needed for effective emergency online teaching. This finding suggests that higher education institutions need to help faculty cultivate innovativeness, technology acceptance, and skill development in order to facilitate a successful conversion to online teaching—especially in the midst of a global pandemic. In particular, such institutions need to develop upheaval strategies that include acknowledging crisis, accepting the responsibility of university leadership, conducting an honest self-appraisal, delineating institutional problems, obtaining material, financial and emotional help, adopting exemplary models, creating situation-specific institutional flexibility, and obtaining freedom from institutional constraints (Diamond, 2019).

The present study yields several theoretical and practical implications. Theoretically, planned voluntary change and urgent inevitable change may be radically different, suggesting that studies on these two topics should have different research agendas. While the former may be concerned with factors that induce change, the latter should focus more on the quality, or depth of change and related influencing factors. The current lack of theoretical guidance for the latter line of research calls for new theories or models that specifically address such forced instructional changes in response to emergencies or crises in higher education such as the COVID-19 pandemic we find ourselves facing today.

In higher education, institutional intervention in response to a crisis can be implemented in two major ways: demonstrating leadership in strategically coping with a crisis by engaging faculty members in planning and then designing faculty development programs to promote innovativeness. Because information communications and technology (ICT) governance is growing in importance as an alternative to face-to-face teaching (especially when it is not feasible, as during the COVID-19 pandemic), universities need to incorporate an ICT center in their main governance system in order to support technology acceptance by faculty and improve the fidelity of instructional supports.

The data used in this study were collected at the initial stage of the pandemic, between May 2020 and July 2020. Therefore, the results may not reflect phenomena that occurred at later stages of the crisis. This time specificity suggests the need for well-designed prospective investigations that address the shifting patterns of instructional changes over longer periods of time. The self-reported method applied in this study may not be sensitive enough to capture faculty beliefs or subsequent changes in their beliefs. To address this issue, a future investigation is needed applying alternative qualitative and quantitative methods, such as phenomenological analysis and beliefs inventory. In addition, this study excluded two of Bronfenbrenner’s ecological systems; exo-system and macro-system. Future research that explores the impacts of factors within these two systems may offer a broader picture of the correlates of instructional changes by university faculty. Nevertheless, this study offers a fresh new perspective that enriches our understanding of individual, course, and institutional factors affecting instructional changes by faculty during the COVID-19 pandemic, and in so doing contributes to universities’ efforts to cope strategically with crises and to ultimately improve the quality of higher education in any context.

## Conclusion

The results of this study suggest that, in higher education contexts, crisis-driven changes may happen differently from pre-planned, voluntary change, and that factors influencing crisis-driven changes are different from those influencing voluntary changes; as reported in previous studies based on technology acceptance theories and models. More specifically, individual faculty members’ progressiveness in technology adoption and innovation and the institution’s reliable and well-managed support for faculty may be more important for crisis-driven change processes, while environmental factors such as social and cultural influences and facilitating conditions are more important in non-crisis contexts. This study offers implications for guiding higher education institutions in developing effective faculty development programs and institutional-level policies and strategies for quality online teaching in crisis situations.

## Data Availability

The data that support the findings of this study are available on request.
